# Acute pancreatitis and subdural haematoma in a patient with severe falciparum malaria: Case report and review of literature

**DOI:** 10.1186/1475-2875-7-97

**Published:** 2008-05-30

**Authors:** Pratibha Seshadri, Anand Vimal Dev, Surekha Viggeswarpu, Sowmya Sathyendra, John Victor Peter

**Affiliations:** 1 Medical Intensive Care Unit, Christian Medical College & Hospital, Vellore 632 004, India

## Abstract

*Plasmodium falciparum *infection is known to be associated with a spectrum of systemic complications ranging from mild and self-limiting to life-threatening. This case report illustrates a patient who had a protracted course in hospital due to several rare complications of falciparum malaria. A 21-year old man presented with a five-day history of high-grade fever, jaundice and abdominal pain and a two-day history of altered conscious state. A diagnosis of severe falciparum malaria was made based on the clinical presentation and a positive blood smear with parasitaemia of 45%. Despite adequate anti-malarial therapy with artesunate, the patient had persistent and worsening abdominal pain. Investigations suggested a diagnosis of acute pancreatitis, a rare association with falciparum malaria. However, in spite of supportive therapy for acute pancreatitis and a 10-day course of intravenous artesunate and oral doxycycline at recommended doses, he continued to be febrile with peripheral blood smear showing persistence of ring forms. Antimalarial therapy was, therefore, changed to quinine on the suspicion of possible artesunate resistance. On the 17^th ^day of stay in hospital, the patient developed generalized tonic-clonic seizures. Computerized tomography of the brain showed bilateral fronto-parietal subdural haematomas that were surgically drained. His fever persisted beyond 30-days despite broad-spectrum antibiotics, quinine therapy and negative malarial smears. A possibility of drug fever was considered and all drugs were ceased. He subsequently became afebrile and was discharged on the 38^th ^hospital admission day. Recognition of complications and appropriate management at each stage facilitated successful outcome. This report has been presented to highlight the occurrence of several rare complications of falciparum malaria in the same patient.

## Background

Falciparum malaria is a common disorder in the tropics associated with myriad complications that can often be life-threatening and fatal [1]. A 21 year old man presented with a mixed infection with falciparum and vivax malaria. His course in hospital was complicated by the development of acute pancreatitis, bilateral subdural haematomas and probable drug fever and artesunate resistance.

## Case presentation

A 21-year-old male, a mechanic by profession, presented to the accident and emergency department with a 5-day history of fever, jaundice and generalized abdominal pain and a 2-day history of altered conscious state. There was no significant past medical history nor was there a history of substance abuse.

**Figure 1:**
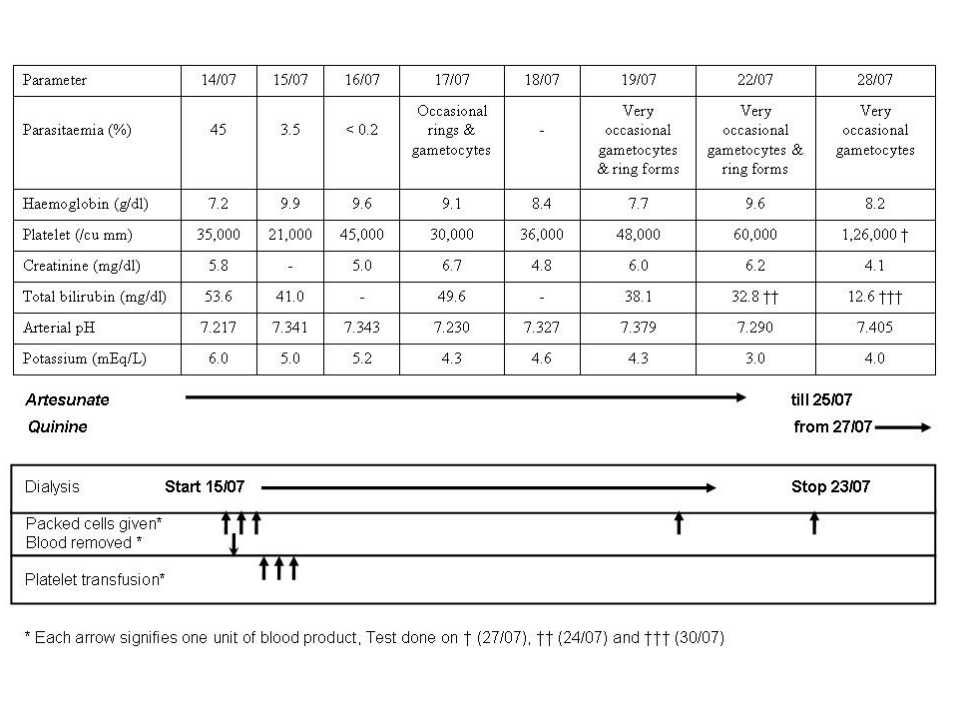
Sequential laboratory parameters of the patient over time.

Clinical examination at the time of presentation revealed a young man who was drowsy but arousable, conscious and oriented. He was febrile (101.5°F), had icterus and exhibited a flapping tremor. He was hemodynamically stable. Systemic examination was unremarkable except for generalized tenderness and mild hepatosplenomegaly on abdominal examination. Investigations on admission are summarized in Table 1. Of note were anaemia (Hb 7.2 gm/dl), thrombocytopaenia (35,000/cumm), renal failure (serum creatinine 5.8 mg/dl) and marked hyperbilirubinaemia (total bilirubin 53.6 mg/dl). Peripheral blood smear was positive for *Plasmodium falciparum *and *Plasmodium vivax *gametocytes and ring stages, with marked parasitaemia of 45%. A diagnosis of severe falciparum malaria was made and treatment was initiated with intravenous artesunate (140 mg at admission and at 12 hours, dose calculated based on an estimated weight of 65 kg) and oral doxycycline therapy (100-mg twice daily) in the emergency department. The dose of artesunate was increased to 180 mg intravenously daily in the intensive care unit. Broad-spectrum antibiotics (intravenous cefepime 1-gm twice daily) were added to cover for a co-existent gram negative sepsis. In view of the high parasitic index and severe anaemia at presentation, three units of packed red cells were administered to the patient and 250 ml of blood removed from the patient in a partial exchange transfusion. A complete ml to ml exchange transfusion was not performed as the haemoglobin value reduced after the first exchange to 5.2 g%. The three units of packed cells and the partial exchange transfusion reduced the parasitaemia from 45% at admission to 3.5% in 24 hours (Figure 1).

**Table 1:** Laboratory data

Variable	Value	Reference range
**Haematologic**		
Haemoglobin (g %)	7.2	10.0 – 15.5
White-cell count (/cu mm)	9100	4000 – 11000
Neutrophils (%)	52	1.8 – 7.5
Platelets (/cu mm)	35000	150000 – 400000
International normalized ratio	1.18	0.8 – 1.2
Activated Partial thromboplastin time (sec)	36.7	23.8 – 37.4
		
**Biochemical**		
Sodium (mmol/l)	124	137 – 145
Potassium (mmol/l)	6	3.5 – 5.0
Bicarbonate (mmol/l)	8	22 – 29
Blood urea (mmol/l)	9	2.7 – 8.0
Creatinine (mg%)	5.8	0.5–1.4
Total Bilirubin (mg%)	53.6	0.5–1.4
Direct Bilirubin(mg%)	23.0	
Albumin (g%)	2.8	3.5–5.0
AST (U/L)	197	8–40
ALT (U/l)	101	5–35
ALP (U/l)	112	40–125
		
**Arterial blood gas (on FiO**_2_**of 0.21)**		
pH	7.22	7.35 – 7.45
PaO_2 _(mm Hg)	187	60 – 90
PaCO_2 _(mm Hg)	35.9	35 – 45
Bicarbonate (mmol/l)	14	22 – 29
Base Excess	-12.8	-2 to 2

**Figure 2:**
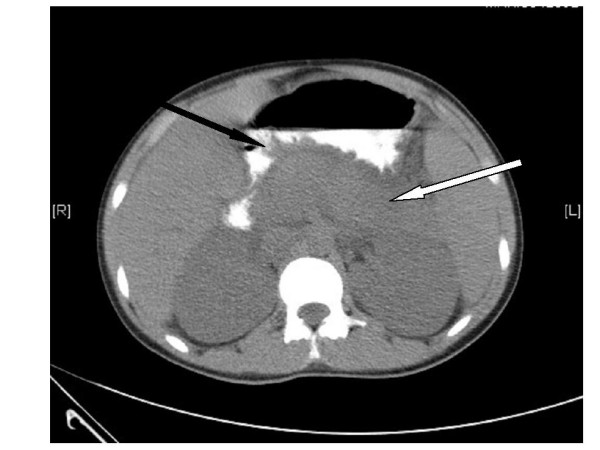
Non contrast computed tomography (CT) scan of the abdomen showing a bulky pancreas (white arrow) with peri-pancreatic fat stranding (black arrow) suggestive of acute pancreatitis.

He was admitted to the intensive care unit (ICU) for treatment and monitoring. In the ICU, his acute renal failure was initially managed conservatively by achieving adequate filling pressures (central venous pressure), maintenance of blood pressure and measures to correct hyperkalemia. However, in view of persistent renal failure and hyperkaliaemia that were refractory to medical therapy, dialysis was initiated and continued till renal recovery. On the second day following ICU admission, his abdominal pain worsened with the development of abdominal guarding and rigidity. Investigations suggested a diagnosis of acute pancreatitis with serum amylase and lipase levels of 1712 U/L (Reference range 0–200 U/L) and 5217 U/L (reference range 0–190 U/L) respectively. A non-contrast computed tomography (CT) scan of the abdomen showed bulky pancreas with significant peri-pancreatic fat stranding (Figure 2). C-reactive protein was > 190 (Reference range-0–6 mg/L). There was no evidence of a hollow viscus perforation.

The acute pancreatitis was managed with analgesics, hydration and supportive therapy. A naso-jejunal tube was inserted to facilitate nutritional supplementation and antibiotics were changed to imipenem in view of severe pancreatitis. However, fever persisted even after ten days of artesunate therapy with the presence of ring forms of falciparum malaria (parasitemia not quantifiable) on the peripheral smear. He was initiated on intravenous quinine therapy on the suspicion of artesunate resistance/failure. Following initiation of quinine therapy, the ring forms were not demonstrable on the peripheral smear. His course was further complicated by urinary tract infection, with > 100000 colonies/ml of *Escherichia coli *and *Pseudomonas aeruginosa *grown from a catheter specimen of urine.

**Figure 3:**
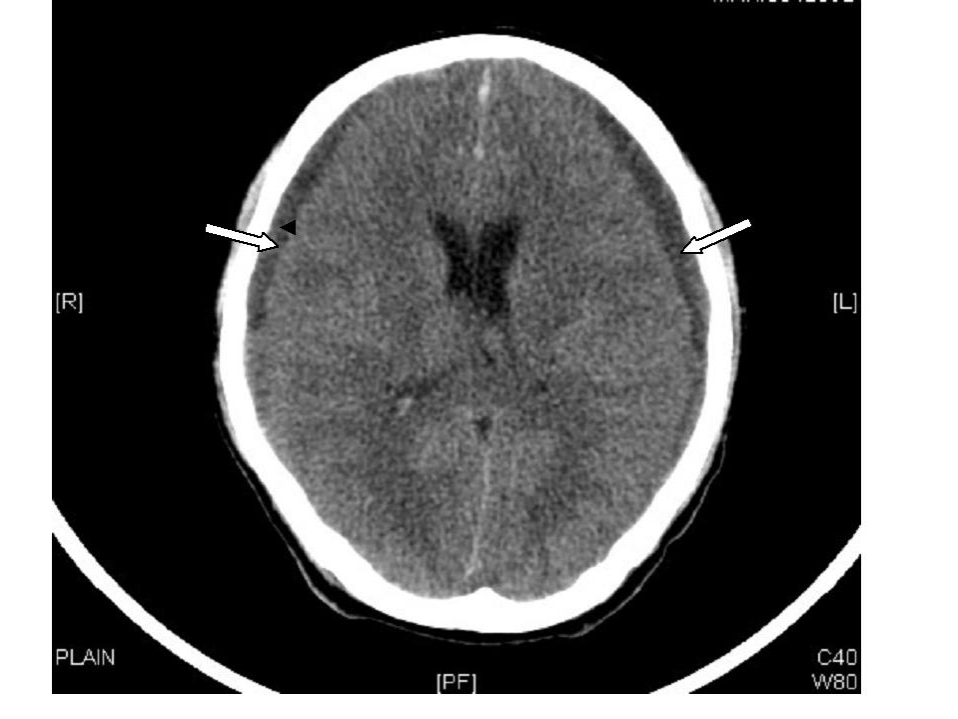
Plain computed tomography (CT) scan of the head showing bilateral subdural haematomas (marked by white arrows).

On Day 17, the patient developed one episode of generalized tonic clonic seizures with post-ictal drowsiness. A CT scan of the brain showed features of acute bi fronto-temporal subdural haematoma with no mass effect (Figure 3). He was initiated on gabapentin 300 mg twice daily (subsequently increased to 300 mg thrice daily), in addition to clobazam 10 mg once a day, at the advice of the neurologist. The subdural haematomas were surgically drained by the neurosurgeons. His consciousness improved and he had no further seizures.

However, his fever did not resolve despite appropriate anti-biotic therapy. Search for a cause for fever including nosocomial infections and sepsis were negative. A diagnosis of drug fever was proposed and all medications were ceased. After 38-days of admission, on becoming afebrile, and free of ring stages on peripheral smear, he was discharged on a fourteen-day course of primaquine.

## Discussion

The occurrence of several rare complications in the same patient is not common. Although abdominal pain is a frequent symptom in malaria, in this patient, the persistent and worsening abdominal pain was related to severe pancreatitis. Abdominal pain and vomiting are reported to be more often associated with falciparum infections than with vivax [1]. The causes of abdominal pain in malaria are protean (Table 2) and include acalculous cholecystitis [2], acute surgical abdomen [3], splenic rupture [4], splenic infarction [5], splenic torsion [6] and hepatitis/hepatomegaly [1]. Abdominal pain has also been described as part of acute renal failure and algid malaria [7]. The presence of abdominal pain and hepatomegaly has been shown to be associated with a higher mortality [7].

**Table 2:** Causes of abdominal pain in acute severe malaria

Organ	Manifestation [reference number]
Abdomen (general)	Acute surgical abdomen [3]
Spleen	Splenic rupture [4]
	Splenic infarction [5]
	Splenic torsion [6]
Liver	Hepatomegaly [1]
	Hepatitis [1]
Gall bladder	Acute acalculous cholecystitis [2]
Renal	Acute renal failure [7]

Pancreatitis is an unusual cause of abdominal pain in malaria with less than 10 cases reported worldwide. There have been isolated reports of a 17-year-old man [8], a 26-year old man [9], a 40-year old man [10] and a 58-year old woman [10] with falciparum malaria and pancreatitis. The parasitaemia in three of these patients [8,10] were low (0.5 to 1.5%). None of these patients had more than one WHO criteria for severe falciparum malaria. There has also been a previous report of a 19-year old girl with acute pancreatitis and adult respiratory distress syndrome in association with malaria [11]. This young man did not manifest acute respiratory distress syndrome either with severe malaria or with acute pancreatitis.

The proposed pathophysiological mechanisms that result in pancreatitis are capillary blockage by parasitized RBCs and acute haemolysis [10]. Occlusion of blood vessels of the pancreas with parasitized RBCs and rosettes have been demonstrated in autopsy studies [8]. There has been no correlation between the infestation index and the occurrence of pancreatitis in these patients [12]. The successful outcomes of patients manifesting pancreatitis in earlier reports and in the patient reported here are encouraging. It is possible that the incidence of pancreatitis is much higher than what is reported and may be due the failure to screen for pancreatitis as a cause of abdominal pain in these patients. It should also be remembered that an isolated increase in lipase levels may not by itself suggest pancreatitis, particularly in the critically ill patient [13]. In a recent study, critically ill patients who had a positive finding on imaging had mean peak lipase levels of 2, 231 ± 715 U/L [13], suggesting that higher lipase level thresholds may need to be used to identify critically ill patients who would benefit from further radiological assessment. The patient in question had a very high lipase level (5217 U/L) and radiological evidence of acute pancreatitis.

The reason for the persistence of parasitaemia on Day 10 was not immediately apparent. The possibilities considered were inadequate dose of anti-malarial therapy, "ineffective" anti-malarial drug, primary drug resistence to artesunate or delayed parasitic clearance due to surgical or functional asplenia. Although it was not possible to get an accurate weight of the patient on admission as he was critically ill, he was estimated to be around 65 kg. His body weight on the 10^th ^hospital day, when he was able to stand, was 50 kg and it was estimated that he had lost about 20% of his body weight during his critical illness. The dose of 140 mg of artesunate administered in the emergency department was thought to be inadequate and this was increased to 180 mg following ICU admission (from the 12-hour dose). The dose regimen of artesunate, of 2.4 mg/kg of artesunate at 0, 12, 24 hours and subsequently daily, administered to the patient based on the SEAQUAMAT trial [14], was considered to be adequate.

In-effective anti-malarial therapy due to poor quality of the drug or fake anti-malarial drugs could have also contributed to persistent parasitaemia. The brand of artesunate bought from the hospital pharmacy by the relatives was from a reputed manufacturer that has its presence in 30 countries with more than 300 registrations. Although the efficacy of the anti-malarial drug could not be assessed by means of a bio-assay, the absence of lack of efficacy in other patients treated for falciparum malaria at the same time with the same batch of artesunate, suggests that persistence of parasitaemia was unlikely to be due to poor drug quality.

Delayed parasitic clearance has also been reported in splenectomized individuals. In an early report of four splenectomized patients with malaria, the clinical course was found to be uncomplicated in all four patients, with the parasite clearance delayed only in the one nonimmune patient and not in the other three partially immune splenectomized patients [15]. In a subsequent report, delayed parasite clearance was reported in a splenectomized patient with falciparum who was treated with artemisinin derivatives [16]. In a more recent publication, two splenectomized individuals had persistence of parasitaemia despite appropriate treatment and adequate absorption with one patient not surviving the illness [17]. In this study both the patients were immune with a moderate to high level of antibodies to *P. falciparum*. It is thus unclear if splenectomy alone increases the risk of delayed parasite clearance or if it needs to be associated with a low immune or non-immune state. The patient described in this case-report had an enlarged spleen (13.3 cm) on ultrasound examination. There were no Howell-Jolly bodies on the peripheral smear to suggest functional asplenia, although their counting is not considered a reliable measure of splenic function [18].

Thus, persistence of parasitaemia in the patient reported in this study is likely to be due to failure of intravenous artesunate therapy. Whilst assessment of genetic polymorphism and inhibitory concentration of 50% (IC_50_) for artemether would have been contributory to the assessment of recrudescence, these could not be performed for the patient presented in this case report.

Artemisinin resistance however is a contentious issue [19]. In a retrospective study of 104 malaria patients, 32 of whom recrudesced, there were no differences in *in vitro *artesunate sensitivities between 6 non-recrudescent isolates and 16 paired admission and recrudescent isolates [12]. The authors suggested that recrudescence with artemisinin therapy was due to parasite density and not due to inherent parasite resistance [12]. Other studies have however suggested that genes that encode transport proteins may be involved in drug resistance.

The commonly implicated mutations in drug resistance in malaria are the *pfmdr1 *(*Plasmodium falciparum *multidrug resistance gene 1) and *pfcrt *(*Plasmodium falciparum *chloroquine resistance transporter gene) genes [20]. An initial study of 115 patients suggested that *pfmdr1 *gene was not associated with treatment outcomes in patients treated either with mefloquine or a combination of mefloquine and artesunate [21]. However, more recently, an increase in the *pfmdr1 *copy number was found to be associated with up to a 40-fold decrease in the *in vitro *susceptibility to mefloquine [20]. In the same study, a combination of mefloquine-artemisinin was associated with increased *pfmdr1*copy number through a 25-fold range of IC_50 _values [20]. The presence of parasites with three or more copies of *pfmdr1 *before treatment, in another study, was again strongly associated with recrudescence [22], whilst decreasing *pfmdr1 *copy number heightened susceptibility to antimalarials [23]. It is now being suggested that polymorphisms in the gene encoding the sarco-endoplasmic reticulum Ca^2+^-ATPase (SERCA) *PfATP6 *gene may be associated with in vitro resistance to artemether in field isolates of *P falciparum *[19].

It is again not clear as to why the patient reported in this study failed to respond to doxycycline, added to the initial treatment regime. Doxycycline has been recommended as an alternative agent for the treatment [24] and prophylaxis of malaria [25] as well as adjunct therapy [26]. The addition of doxycycline to a regimen of sulphadoxine/pyrimethamine was associated with an improvement in the cure rates from 78% to over 90% [26]. However, pharmacokinetic studies suggest that the dosing of 200 mg per day may not be optimal [27]. Further, it is well recognized that oral absorption of drugs is significantly reduced in the critically ill patient. It is likely that reduced absorption of orally (nasogastrically) administered doxycycline in sub-optimal doses in this critically ill patient may have possibly contributed to the lack of response.

The occurrence of intracerebral haemorrhage or haematoma is again a rare complication. The mechanism of development of subdural haematoma in the patient reported in this study was not evident. Although the family denied history of trauma, this could not be ruled out with certainty. It is likely that his subdural haematoma was at least in part contributed by the thrombocytopenia. However symptoms became evident in this patient much after the platelet count started improving.

In a previous report, a three-year old girl with anaemia, thrombocytopaenia and positive blood smear for falciparum malaria was diagnosed to have a spontaneous subdural empyema [28]. This child was treated with surgical evacuation, intravenous quinine and antibiotics [28]. In another report, extensive subarachnoid haemorrhage was described in a 54-year old woman that returned from Kenya [29]. Focal haemorrhage has also been reported in the left front falx of a patient diagnosed to have malaria [30]. This is probably the first case of bilateral (spontaneous) subdural haematoma in association with falciparum malaria.

## Conclusion

Despite several strategies for malaria prevention and control, malaria continues to pose several public health challenges in the developing world. This case has been reported to illustrate the occurrence of several rare and alarming complications in one patient. An open and inquisitive approach to the multiple symptoms of patients presenting with malaria would enable the prompt recognition of these complications and a successful outcome.

## Authors' contributions

JVP and SV conceived study, All authors participated in the co-ordination and helped to draft the manuscript, PS, AVD and JVP were involved in literature search and literature review. All authors read and approved the final manuscript.

## Conflict of interest

The authors declare that they have no competing interests.
